# The Intricate Network of Adipokines and Stroke

**DOI:** 10.1155/2015/967698

**Published:** 2015-12-10

**Authors:** Ema Kantorová, Ľubica Jesenská, Daniel Čierny, Kamil Zeleňák, Štefan Sivák, Matej Stančík, Peter Galajda, Vladimír Nosáľ, Egon Kurča

**Affiliations:** ^1^Jessenius Faculty of Medicine, Comenius University, Clinic of Neurology, Malá Hora 4A, 03601 Martin, Slovakia; ^2^Jessenius Faculty of Medicine, Comenius University, Department of Medical Biochemistry, Malá Hora 4A, 03601 Martin, Slovakia; ^3^Jessenius Faculty of Medicine, Comenius University, Clinic of Radiodiagnostics, Malá Hora 4A, 03601 Martin, Slovakia; ^4^Jessenius Faculty of Medicine, Comenius University, Clinic of Internal Medicine I, Malá Hora 4A, 036 01 Martin, Slovakia

## Abstract

Cerebrovascular disorders, particularly ischemic stroke, are one of the most common neurological disorders. High rates of overweight and obesity support an interest in the role of adipose tissue and adipose tissue releasing cytokines in inducing associated comorbidities. Adipokines can serve as a key messenger to central energy homeostasis and metabolic homeostasis. They can contribute to the crosstalk between adipose tissue and brain. However recent research has offered ambiguous data on the network of adipose tissue, adipokines, and vascular disorders. In our paper we provide a critical insight into the role of adipokines in evolution of ischemic stroke.

## 1. Introduction

Adipose tissue is a highly specialized organ that stores excess energy and releases it when needed by other tissues [1]. Obesity develops when the intake of calories exceeds energy expenditure [1]. Depending on the time of onset, obesity is associated with increased adipocyte size and number [1]. Further, an increase in adiposity results in fat accumulation in depots with different metabolic properties. Visceral fat has a higher risk for development of metabolic diseases, whereas subcutaneous fat does not [2].

Adipose tissue is an active secretory organ that elaborates a variety of messenger molecules. Adipocyte-derived hormones have structural homology to cytokines that actively participate in regulating many biological processes [3]. Major target receptors for the messenger molecules are located in adipose tissue [4]. They are also found in the hypothalamus, skeletal muscle, and liver [1]. Adipokines can also exert endocrine effects and participate in an interplay between several tissues [4].

It is still not fully clear whether adipokines are obesity-dependent or obesity-independent risk factors for neurological diseases and how they coordinately regulate physiological functions. However, the link between obesity-associated systemic inflammation and cerebrovascular [3], autoimmune [5], and degenerative neurological disorders has become of major interest [6, 7].

## 2. Ischemic Stroke and Obesity

Stroke, a leading course of death or disability, shares many risk factors with cardiovascular diseases (CVD), such as age, smoking, hypertension, diabetes mellitus, inactivity, overweight or obesity, and dyslipidemia [1]. Their role in ischemic stroke (IS) is still less elucidated than for CVD [8]. Some studies have suggested that obesity is an independent risk factor for cerebrovascular diseases [8–12]. The measures of obesity include body mass index (BMI), waist-to-hip ratio, and waist circumference (WC). BMI helps to classify rate of excess adipose tissue as overweight when BMI is >25 kg/m^2^ and as obesity when BMI > 30 kg/m^2^ [8, 9]. Study results have not always been conclusive and have often been contradictive. For example, markers of abdominal adiposity showed a graded and significant association with risk of IS, independent of other vascular risk factors. It was reported that for cerebrovascular events predictive function of WC is better than the one of BMI [12]. On the other hand one of the studies recorded higher BMI, especially BMI > 30 kg/m^2^, in male subjects, to correlate with an increased risk of cerebrovascular accidents [13]. Controversially, Wannamethee and colleagues found BMI and WC not to be associated with risk of IS, showing the lowest risk of IS in obese men (BMI > 30 kg/m^2^) [14]. The findings were consistent with previously published studies that showed no association of obesity and IS in older men [15, 16]. The phenomenon called “obesity paradox,” described especially in older patients, may reflect changes of body morphology in the elderly [14].

The correlation of excessive body weight and carotid artery disease indicates a role of obesity in development of IS [17]. The diameter and stiffness of carotid arteries appear to increase with higher BMI. Carotid distensibility decreases with higher BMI more at young than at old age. In elastic arteries, the relationship between arterial stiffness and BMI is more complex and varied with gender and age [17].

Excess adipose tissue is responsible for a wide variety of released adipokines such as leptin, adiponectin, resistin, visfatin, and apelin that have been associated with cerebrovascular diseases [18]. Now we are going to discuss those important adipokines that are considered to be associated with ischemic stroke.

## 3. Adipokines and Ischemic Stroke

### 3.1. Leptin

Leptin (LEP) is expressed mostly in adipose tissue, although low levels were found in other organs [19]. It circulates as both a bound and a free hormone, the latter representing the bioavailable hormone. LEP concentration is dependent on the quantity of stored energy fat, as well as the status of energy balance. For example, plasma leptin is higher in obese than in lean individuals, falls rapidly during fasting, and increases after feeding. Leptin acts primarily in the brain, bounding specific receptors in the lateral hypothalamus [19].

LEP resistance appears to be a mechanism that is a part of the burden of obesity on health that extends across multiple organ systems. LEP resistance is characterized by decreased availability of LEP to the brain despite normal or even higher plasmatic and cerebrospinal fluid (CSF) levels and peripheral activity. The high levels would be expected to depress appetite but fail to do so [20]. Mechanisms of leptin resistance are complex including genetic mutation of leptin receptor, failure of self-regulation of hypothalamic centers, limited transport of leptin through blood-brain barrier, and intracellular molecular mechanisms [21].

A Finnish twin study established that a substantial percentage of variance in intensity of relationship between hyperleptinemia and vascular complications is attributable to genetic background [22]. According to the study results, genetic effects contributed 72% of total variance in men and 66.4% in nonpregnant women, while 27.8% of variations among men and 33.6% among women were due to environmental effects [22]. In particular important environmental factors are dietary habits, high-fat diet, and overeating with subsequent obesity. In another study both obesity and increased plasma leptin concentration correlated with high blood pressure [23]. Both male and female ischemic stroke patients had higher blood pressure than control subjects [24]. In association with high blood pressure hyperleptinemia in men was found to predict both ischemic and hemorrhagic stroke subtypes [25]. Hyperleptinemia was reported to be a good predictor of uncontrollable high blood pressure [25]. In obese individuals leptin-mediated platelet activation represented a direct link between leptin and the risk of thrombotic complications [26]. A relationship between several coagulation factors (factor VIIa and von Willebrand) and leptin levels supported a role of LEP in procoagulation states frequently associated with vascular complications [27].

Signore and colleagues showed that triglycerides (TG) are capable of reducing leptin transport across the blood brain barrier and modulate function of hypothalamic centers. Therefore the presence of higher plasma triglyceride levels in obese individuals is thought to be directly responsible for the lack of brain LEP concentration and a LEP resistance [20]. Association of higher plasma triglycerides (TG > 2,0 g/L, resp., > 2,8 g/L) with ischemic stroke was also proved by a large Multi-Risk Factor Interventional Trial [28]. The study involving multiethnical patients showed the correlation between hyperlipidemia and ischemic stroke, particularly in women. Considering ethnical differences, White, Afro-American, and Hispanic women had higher levels of TG and both low-density cholesterol (LDL) and high-density cholesterol (HDL) than men with ischemic stroke [28]. The benefits of having higher HDL were seen only in Asian stroke women [28]. A systematic review of epidemiological studies revealed a positive association between elevated TG levels and increased risk of stroke. The study showed a need for new large prospective studies, especially in stroke subtypes [29]. In contrast, in our assessment, LEP correlated with neither TG nor HDL in stroke patients (male and female) [30].

LEP resistance is most often associated with impaired insulin signaling [31] or insulin resistance in patients with IS [32]. Leptin is supposed to promote hyperglycemia-dependent endothelial dysfunction [33–36].

Several population-based studies have reported strong positive association of increased plasma LEP levels with pathogenesis of IS [34, 36–38]. High leptin was significantly and independently associated with IS in both men and women [35, 38]. In our assessment LEP was also reported to be a stroke risk factor for both men and women, with levels found to be three times higher in women than in men with ischemic stroke, but still higher than in controls [30]. Other authors showed that there are sex-differences in leptin activity. Increased LEP was found to be associated with significantly increased risk of ischemic stroke in older men after adjustment for age and BMI [14]. These results originate from a large prospective population study of British men aged 60–79 with no previous diagnosis of myocardial infarction, heart failure, or stroke, in which no women were included [14]. In a case-nested study Söderberg et al. reported that high leptin predicted stroke in men but not in women independently of traditional risks [24]. In a large population-based study, LEP levels were higher in women than men, showing an increased risk of nearly twofold for stroke in women when compared to men [35]. An analysis from another population-based study demonstrated that LEP is a risk factor of stroke in African-American women, independently of age, smoking, obesity status, and hypertension. The differences in leptin signaling pathways in the central nervous system were considered to be based on a sex-specific relationship between leptin and insulin resistance [32]. In female patients severe obesity coexisted with insulin resistance.

In ischemic stroke patients intracerebral LEP deficiency caused by leptin resistance can trigger atherosclerotic/inflammatory vascular changes and vascular stiffness [39]. The study assessed arterial stiffness by carotid-femoral pulse wave velocity. It suggested that leptin represents the relationship between abdominal adiposity and arterial stiffness [39]. The correlation between leptin and intima-media thickness (IMT) was demonstrated in both men and women, independently of age and other vascular risks [40]. The leptin/adiponectin ratio could be a good predictor of IMT, as noted by Norata et al. [41]. On the other hand Gardener and colleagues have found adiponectin to be a better predictor of arterial stiffness than LEP, being inversely associated with IMT after adjustment for demographics and other vascular risks. Moreover, the relationship between adiponectin and IMT appears to be stronger among patients with diabetes [42].

Knowledge of LEP resistance, absence of its central brain activity, and consequences of LEP resistance stimulated researchers to assess LEP as a potential medicament. Signore and colleagues reported potential neuroprotective role of LEP after its intracerebral application [20]. Experimental treatment by leptin has been evaluated in animal models [43]. In mice intraperitoneal administration of leptin was found to decrease infarction volume following middle cerebral artery occlusion [43]. Leptin protection was dose-dependent and remained effective when leptin administration was delayed up to 90 min after the onset of reperfusion by recombinant tissue plasminogen-activator (rtPA). LEP was demonstrated to exhibit neuroprotective effects against ischemic stroke [43]. It seemed promising to use leptin in tandem with rtPA, administering it following rtPA treatment. In the experiment dual treatment extended the time window of efficacy for rtPA treatment and subsequently reduced reperfusion injury [43]. In another study investigation of potential role of leptin in acute IS contradicted animal studies results. Increased plasma LEP levels correlated with final larger infarction volume and leptin was evaluated as harmful in IS development, potentiated by insulin resistance. LEP efficacy was not confirmed in stroke patients treated by rtPA [33]. Further studies are required to elucidate treatment approaches by leptin in humans.

Beneficial effects of leptin in obesity and neuroendocrine/metabolic dysfunction were reported only in rare cases of human congenital leptin deficiency [44]. In affected children leptin administration resulted in sustained gradual improvement of insulin sensitivity, dyslipidemia, and weight normalization [44].

Doubled effect of LEP resistance and obesity were reported to play an important role in altered sensitivity to preventive medication. The increased levels of isoprostanes observed in visceral obesity and hyperleptinemia could be involved both in the persistent platelet activation* in vivo* and in the resistance to antiplatelet effects of aspirin [45].

Dietary options are essential for normalization of adipoendocrinal status. Restriction of leptin resistance and sensitization of leptin receptors are based on weight reduction, insulin sensitivity reactivation, and lipid profile improvement via dietary and medicament management. Fenofibrate was published to depress leptin levels [46].

### 3.2. Adiponectin

Adiponectin (ADI) is one of the most abundant adipokines produced by adipocytes. ADI performs a fundamental role in vascular physiology by modulating the crosstalk between endothelial cells, smooth muscle cells, leukocytes, and platelets [47]. Apart from maintaining vascular homeostasis, ADI also seems to protect from vascular injury and atherogenesis [47, 48]. Since 2001, ADI has attracted much attention because of its potential antidiabetic and anti-inflammatory activities and its potential role as a plasma biomarker of metabolic syndrome [49]. In endothelial cells, ADI was shown to increase nitric oxide (NO) production and improve endothelial-dependent vasodilation [4]. ADI was reported to suppress tumor necrosis factor *α*- (TNF*α*-) induced production of proinflammatory chemokines and adhesion molecules and inhibit cell proliferation [47]. ADI levels were found to be inversely associated with inflammatory markers [50]. ADI in circulation were decreased in obese subjects, with strongly negative correlation between plasma ADI, BMI, and total fat mass [47].

ADI appears to attenuate secretory cytokine profile of blood-brain barrier cells [51]. ADI receptors were found to be expressed on endothelial vascular cells in brain [48]. Since adiponectin may protect the endothelium from early atherosclerotic events such as the expression of adhesion molecules or the attachment of monocytic cells, hypoadiponectinemia could be linked to endothelial damage [52]. Shimabukuro et al. showed lower adiponectin levels closely related to endothelial dysfunction measured by forearm blood flow, proportionally to the severity of obesity. They considered hypoadiponectinemia to enhance endothelial dysfunction and predict future cerebro- and cardiovascular diseases [53].

Hypoadiponectinemia was associated with stroke in patients with advanced intracranial atherosclerosis, especially in men [30, 54]. Obesity-dependent hypoadiponectinemia was associated with increased common carotid IMT in young and middle-aged women [54]. Lo and colleagues reported traditional risk factors of atherosclerosis such as age, diastolic blood pressure, and triglyceride levels to be significantly associated with carotid IMT. In multivariate modeling adiponectin, age, smoking, and subcutaneous abdominal fat were also significantly related to IMT in healthy women across a range of weights. The study considered ADI associated with IMT to be a novel predictive factor for future stroke [55].

Both lower adiponectin and higher leptin showed significant associations with increased frequency of atherothrombotic (large-artery) stroke [54, 56]. ADI levels were found to be highest in cardioembolic stroke patients and lowest in intracranial atherothrombotic stroke groups [30, 54]. Patients with advanced intracranial atherosclerosis defined by ≥1 additional lesion outside the symptomatic arterial territory had lower ADI levels than those with isolated intracranial atherosclerosis [54]. Chen and colleagues showed plasma adiponectin to be significantly lower in ischemic stroke patients than in healthy subjects. According to the authors ADI level remains an independent stroke risk factor [57]. They did not find differences of plasma ADI levels between patients with small- and large-artery infarction [57]. Similarly both extracranial atherothrombotic stroke and small-artery stroke patients have displayed the same levels of plasma ADI [54]. Both these results are partly in contrast with our finding. Our stroke patients showed evidently lower levels of ADI than controls, and the lowest levels were found in men with atherothrombotic stroke and in women with small-artery stroke [30].

However, several studies found no relationship of ADI levels and IS incidence in both older women [58] and men [14]. Rajpathak and colleagues also found circulating levels of ADI not to be independently associated with an increased risk of IS in postmenopausal women. In these patients ADI levels were dependent on obesity and other cardiovascular disease risk factors [59]. However, in another study, increased levels of plasma ADI correlated with increased risk of incident IS among African-American women. Adiponectin levels were significantly higher among the stroke participants with coronary heart disease compared to those without it. Harmful ADI properties have been suggested to be caused by mechanism of “adiponectin resistance” similar to leptin resistance [37]. Confusion in the literature partly relates to complexities in interpreting benefits of higher levels of adiponectin versus its pathological increase as in heart failure [60]. An alternative explanation sees adiponectin overproduced in response to vascular inflammation to counter the atherosclerotic process in arteries [48, 60].

Hypoadiponectinemia can serve as an independent predictor of mortality after IS. Efstathiou et al. reported low plasma ADI to be related to an increased risk of 5-year mortality after first-ever ischemic stroke, independently of other adverse predictors [50]. Plasma ADI levels were found to be positively associated with age, despite higher frequency of vascular risk factors in older patients [61, 62]. It has been suggested that aging and advanced stages of cardio- and cerebrovascular diseases may trigger a counterregulatory response that raises plasma ADI [60].

Carnevale and colleagues reported an inverse relationship between serum ADI and CHA2DS2-VASc in anticoagulated patients with atrial fibrillation, when CHA2DS2-VASc score determines stroke risk for patients with atrial fibrillation. Atrial fibrillation is burdened by enhanced systemic inflammation and platelet activation. In the study it was documented by increased blood levels of soluble proinflammatory marker, a CD40-receptor ligand (CD40L), and low levels of ADI even after administration of anticoagulants [63]. Low levels of ADI in patients with atrial fibrillation suggested a role of ADI to favor platelet activation* in vivo* [63].

According to other reports, lower baseline ADI concentrations inversely correlated with poor outcomes of IS independently of other adverse predictors [50]. All differences between stroke subgroups, stratified according to adiponectin levels, did not reach significance, suggesting relatively weak association of ADI with the etiology of IS [50, 58]. Correlation of reduced adiponectin levels with the studied inflammatory markers (IL-6, IL-18, TNF*α*, and CRP) was not very strong either [60].

The potential beneficial effects of ADI support the rationale for administration of ADI as medication. It has been demonstrated experimentally that the decreased secretion of ADI in obesity alters lipid metabolism and insulin sensitivity in the liver. However, administration of recombinant adiponectin to adiponectin-deficient obese mice fed a high-fat diet dramatically alleviated hepatomegaly, steatosis, and inflammation [64]. Exogenous administration of ADI might counteract the consequences of obesity state and activate its antiatherogenic, vasoprotective, and anticancer actions [65]. Direct supplementation of recombinant ADI in human subjects would be extremely expensive. An alternative approach is to use pharmacological or dietary intervention to enhance ADI actions in target tissues. Thiazolidinediones (rosiglitazone, pioglitazone), inhibitors of angiotensin-converted enzyme, and angiotensin II blockers reinforce positive vascular effects [49, 50]. Statins, thought to improve vascular endothelial functions, have shown ambiguous role when atorvastatin was not proved to decrease ADI levels in diabetic or prediabetic patients [66]. Metformin, a commonly used antidiabetic drug, was shown to mimic the action of ADI and may be potentially used in supplementation of ADI [49]. Other possible treatment targets might be proinflammatory cytokines and chemokines or their receptors, through the use of their agonists or monoclonal antibodies [65].

### 3.3. Resistin

The name resistin (RES) of this adipokine has been derived from the observation that it induced insulin resistance in mice. In contrast, in healthy individuals, comparison of plasma and CSF resistin levels showed a positive correlation of plasma RES with increasing age, but no correlation with BMI or index of insulin resistance (HOMA-IR) [67]. Furthermore, in this study of neurologically intact individuals, CSF resistin levels did not correlate with age or HOMA-IR index, and they remained unaltered by diabetic status [67]. This might be explained by the fact that RES emerges dominantly as a critical mediator of insulin resistance associated with inflammatory settings or sepsis [18]. According to other authors RES could induce similar effects to those of leptin. Increased levels of RES seem to be positively associated with atherosclerosis due to induction of endothelial cells and consequent expression of adhesion molecules, chemokines, and cell proliferation [65, 68]. Resistin can increase the risk of stroke by promoting systemic inflammation and endothelial dysfunction, both playing a significant role in atherosclerosis [68].

Rajpathak et al. found that the association between resistin and IS remained significant after adjustment for obesity as well as markers for inflammation and endothelial dysfunction. The effects of resistin on stroke risk could not be explained by the obesity-associated pathways and might involve additional unidentified biological mechanisms [59]. Resistin is supposed to mediate intensity of ischemic cerebrovascular events. Higher concentrations of resistin and TNF*α* were observed at the first day after IS in female patients after comparing with controls. Follow-up revealed sustained elevation of TNF*α* levels and RES on the 10th day after the onset. Resistin positively correlated with TNF*α* and stroke severity [69].

The participation of RES in endothelial dysfunction in insulin-resistant patients related to its direct effect on endothelial cells promoting the release of endothelin-1 [70]. The proliferative effect of RES was suggested to underlie the increased incidence of restenosis after artery stenting common among diabetic patients [70].

### 3.4. Apelin

Apelin (APE) is a bioactive peptide that was originally identified as the endogenous ligand of the orphan G-protein-coupled receptor. In obese mice, increased levels of apelin corresponded with mild inflammation induced by obesity, characterized by an increase in macrocytes count and high TNF*α* levels [71].

In obesity, increased levels in plasma and adipose tissue were reported [65]. APE was associated with a positive hemodynamic profile, having a positive inotropic effect in normal and failing rat hearts [65]. Reduced apelin levels were found in patients affected by single atrial fibrillation and chronic heart failure [72, 73]. Apelin has been recently identified as an angiotensin II homologue with an impact on vasoreactivity [74]. In contrast, APE treatment was reported to have beneficial effect on aortic wall, causing its relaxation [74]. The function of APE in development of cerebrovascular disorders is not fully clear. A recently reported case-control study did not find any differences in apelin plasma levels between IS patients and healthy controls [75]. In humans apelin levels in acute IS were not elevated, contrary to leptin levels [75].

### 3.5. Visfatin

Visfatin (VIS) is a recently discovered adipokine, secreted mainly by visceral adipose tissue [71]. According to other authors, VIS plasma levels were shown to correlate with measures of global obesity but not visceral-fat mass or waist-to-hip ratio [18]. VIS is an insulin-mimetic adipokine that was originally discovered in liver, skeletal muscle, and bone marrow as a growth factor for B lymphocyte precursors. It was upregulated in models of acute lung injury and sepsis. Circulating visfatin levels closely correlated with white adipose tissue accumulation. VIS synthesis seems to be regulated by several factors, including glucocorticoids, TNF*α*, interleukin-6 (IL-6), and growth hormone [71]. Relationship of VIS and type 2 diabetes was also described [18].

Role of VIS in stroke has been studied and reported by several authors. They showed increased plasma VIS levels in acute IS [75, 76]. After adjustment for diabetes, hypertension, dyslipidemia, and age, visfatin was assessed as independent predictor of acute IS [75]. Predictive role of VIS was demonstrated in 6-month follow-up [76].

VIS seems to be a prognostic factor of cardiovascular mortality [75]. Visfatin seems to have a key role in plaque destabilization, associated with its increased expression in macrophages of human unstable carotic and coronary atherosclerosis. In microarray VIS gene was markedly enhanced in carotic plaques in symptomatic individuals compared with plaques in asymptomatic individuals [77]. The relationship between inflammatory markers and VIS levels in patients with symptomatic carotic atherosclerosis supported an inflammatory role of VIS as a mediator in carotic atherosclerosis [77] and future stroke. Higher plasma VIS levels were reported in patients with acute IS associated with symptomatic carotid stenosis (>50%) [75].

## 4. Conclusion

Increase in rates of overweight and obese people in industrial countries has awakened the interest in the role of adipose tissue in metabolic and hormonal balance in the human body. Adipokines, hormones released by adipose tissue, are divided into two groups. One includes those with anti-inflammatory, antidiabetic, and anabolic functions such as adiponectin. The other includes hormones with proinflammatory, prodiabetic, and catabolic functions, for example, leptin, resistin, visfatin, and probably apelin. Now we know that adipokines regulate metabolism via hypothalamic receptors, and they also function as cytokines, being linked to innate immunity. Moreover, adipokines that appear to regulate endovascular compartment can be crucial for later development of arterial stiffness. Increase in adipose tissue disturbs the balance in adipokine production and activity. Participation of certain adipokines in pathomechanisms of ischemic stroke has been proved by independent studies. However, studies have shown them to be both obesity-dependent and obesity-independent factors. It is obvious that there are differences between populations in prevalence of obesity, as well as in diet, calorie intake, and level of physical activity. Interindividual variability of impact of adipokines on target tissues of subjects in high risk of IS poses the question about the role of genetic background of adipocytes' reactivity to environmental influence. Precise mechanism of functioning of adipokines is probably dependent on other not fully known factors. Despite the volume of current information about adipokines, our knowledge is still incomplete and requires further studies. Even so, it is possible to link excessive cumulation of adipose tissue to ischemic stroke risk.

## Abbreviations

ADI: Adiponectin
APE: Apelin
BMI: Body mass index
CD40L: Ligand of a CD40 receptor, expressed by T cells under inflammatory conditions
CRP: C-reactive protein
CSF: Cerebrospinal fluid
CVD: Cardiovascular diseases
HDL: High-density cholesterol
HOMA/IR: Index of insulin resistance
CHA2DS2-VASc: A stroke risk score for patients with atrial fibrillation
IL-6: Interleukin-6
IL-18: Interleukin-18
IMT: Intima/media thickness
IS: Ischemic stroke
LDL: Low-density cholesterol
LEP: Leptin
NO: Nitric oxide
RES: Resistin
TNF*α*: Tumor necrosis factor *α*
TG: Triglycerides
rtPA: Recombinant tissue plasminogen-activator
VIS: Visfatin
WC: Waist circumference.
